# Emerging Foodborne Trematodiasis

**DOI:** 10.3201/eid1110.050614

**Published:** 2005-10

**Authors:** Jennifer Keiser, Jürg Utzinger

**Affiliations:** * Swiss Tropical Institute, Basel, Switzerland

**Keywords:** Foodborne trematodiasis, life cycle, epidemiology, contextual determinants, geographic distribution, population at risk, aquaculture development, perspective

## Abstract

Foodborne trematodiasis is an emerging public health problem, particularly in Southeast Asia and the Western Pacific region. We summarize the complex life cycle of foodborne trematodes and discuss its contextual determinants. Currently, 601.0, 293.8, 91.1, and 79.8 million people are at risk for infection with *Clonorchis sinensis*, *Paragonimus* spp., *Fasciola* spp., and *Opisthorchis* spp., respectively. The relationship between diseases caused by trematodes and proximity of human habitation to suitable freshwater bodies is examined. Residents living near freshwater bodies have a 2.15-fold higher risk (95% confidence interval 1.38–3.36) for infections than persons living farther from the water. Exponential growth of aquaculture may be the most important risk factor for the emergence of foodborne trematodiasis. This is supported by reviewing aquaculture development in countries endemic for foodborne trematodiasis over the past 10–50 years. Future and sustainable control of foodborne trematodiasis is discussed.

## Abstract 2

Foodborne trematodiasis is emerging because of increased aquaculture.

Foodborne trematodiasis, which is caused by liver flukes (*Clonorchis sinensis*, *Fasciola* spp., *Opisthorchis* spp.), lung flukes (*Paragonimus* spp.), and intestinal flukes (*Echinostoma* spp., *Fasciolopsis buski*, heterophyids), is an emerging public health problem. In China, clonorchiasis infections have more than tripled over the past decade; ≈15 million people were infected with *C. sinensis* in 2004 (*1*).

The epidemiology of foodborne trematodiasis has changed in recent years. In some settings, the prevalence of foodborne trematode infections decreased significantly, which can be explained by factors such as social and economic development, urbanization, adequate food inspections, health education campaigns, use of chemical fertilizers, and water pollution (*2**–**5*). In many other areas, however, higher frequencies and transmission dynamics have been observed, which is probably the result of expansion of aquaculture for production of freshwater fish and crustaceans and improved transportation and distribution systems to bring these aquatic foods to local and international markets (*5**,**6*).

The contribution of aquaculture to global fisheries increased from 5.3% in 1970 to 32.2% in 2000 (*7*). By 2030, at least half of the globally consumed fish will likely come from aquaculture farming (*8*). Total global registered aquaculture production in 2000 was 45.7 million tons, of which 91.3% was farmed in Asia (*7*). Freshwater aquaculture production has increased at a particularly high rate; currently, it accounts for 45.1% of the total aquaculture production. For example, the global production of grass carp (*Ctenopharyngodon idellus*), an important species cultured in inland water bodies and a major intermediate host of foodborne trematodes, increased from 10,527 tons in 1950 to >3 million tons in 2002, accounting for 15.6% of global freshwater aquaculture production (http://www.fao.org). The major producer of grass carp is China, where it is traditionally eaten raw as sushi or *yusheng zhou* (*1*).

As the world's population continues to grow, efforts to increase annual fish production are essential to maintain food with a high protein value. To meet the projected demand, global production of aquatic products needs to double over the next 25 years (*9*). Because wild stocks are being increasingly overfished, ≈50% of marine fisheries are being used at maximum capacity, the aquaculture sector must expand to meet future needs (*8**,**9*). Aquaculture production is expected to grow at an annual rate of 5% to 7% at least until 2015 (*10*). Aquaculture development will provide employment and spur economic growth, both important factors for reducing poverty. However, this expansion and intensification of aquaculture should be monitored carefully in countries where foodborne trematodes are endemic because their frequencies might increase, leading to more subclinical and clinical disease.

To our knowledge, no comprehensive analysis of the relationship between occurrence of foodborne trematodiasis and development of water resources has been conducted. This situation motivated us to update estimates of people at risk for the major foodborne trematodes, to quantify the changes in freshwater fish and crustacean production in the past 10–50 years in trematodiasis-endemic countries, and to examine the relationship between proximity of human habitation to freshwater bodies and infections with liver, lung, or intestinal flukes. Our work will contribute to strengthening and expanding the current evidence base of contextual determinants of water-related, vectorborne diseases, including malaria (*11*), lymphatic filariasis (*12*), and Japanese encephalitis (*13*).

## Life Cycle

The complex life cycle of foodborne trematodes has been summarized in recent publications (*1**,**5*). Briefly, parasite eggs from infected humans or animals reach freshwater bodies through contaminated fecal matter, e.g., through nonhygienic defecating habits of humans or the use of human feces for fertilizer (night soil) (*4*). Foodborne trematodes have widespread zoonotic reservoirs. Cats, dogs, foxes, pigs, and rodents are definitive hosts for *C. sinensis*, and domestic ruminants serve as reservoirs for *Fasciola hepatica* infections (*1**,**14*). Once eggs have reached a suitable body of fresh water, they develop and release a miracidium. It enters an aquatic snail, which acts as first intermediate host. Inside the snail, within several weeks, the miracidium transforms into cercariae. They are released into the freshwater environment and attach, penetrate, and encyst as metacercariae in susceptible second intermediate hosts. Infection with foodborne trematodes is accomplished through ingestion of metacercariae by eating raw or insufficiently cooked freshwater fish (*C. sinensis*, *Opisthorchis* spp., *Echinostoma* spp., heterophyids, *Metagonimus* spp.), freshwater crab or crayfish (*Paragonimus* spp.), aquatic plants (*Fasciola* spp., *Fasciolopis buski*), snails or tadpoles (*Echinostoma* spp.), or by drinking contaminated water (*Fasciola* spp.).

## Contextual Determinants

**Figure 1:**
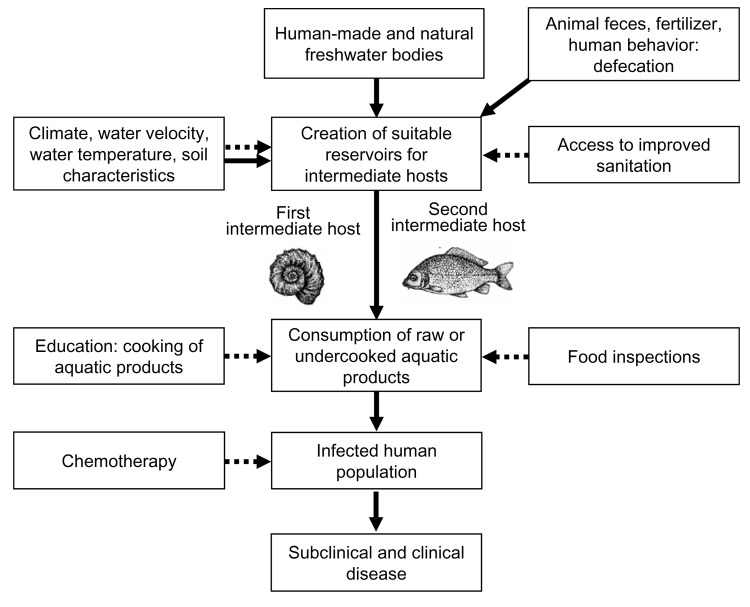
Contextual determinants of foodborne trematodiasis. Solid arrows, negative impact; dashed arrows, positive impact.

Figure 1 depicts the contextual determinants of foodborne trematodiasis. The most important epidemiologic features responsible for transmission of foodborne trematodes include 1) ecologic and environmental factors, 2) behavioral factors, and 3) socioeconomic and cultural factors.

Population dynamics of the first intermediate host snails are affected by several environmental factors, particularly the quality, current, and temperature of the fresh water. For example, *Fossaria cubensis* and *Pseudosuccinea columella*, first intermediate hosts of *F. hepatica*, were studied in Cuba. While the former snail was more abundant in polluted habitats, the latter snail prefers clean water (*15*). Rainfall or evapotranspiration also show a correlation with intermediate host snail populations. In many countries, a seasonal distribution of facioliasis affected by temperature and rainfall has been observed (*16*).

More than 100 fish species are secondary intermediate hosts for *C. sinensis* and 35 are secondary intermediate hosts for *Opisthorchis* spp (*17*). More than 50 species of crustacean have been identified as secondary intermediate hosts for *Paragonimus* spp (*17*). With the exception of the second intermediate host of the heterophyids (mullets, perches, and gobies), which thrive in brackish water (*4*), the second intermediate hosts of the other foodborne trematodes are confined to stagnant or slow-flowing fresh water (Table 1). Irrigation schemes, particularly those for rice growing, are also highly suitable reservoirs for the intermediate hosts.

**Table 1:** Geographic distribution and population at risk for major foodborne trematode infections

Foodborne trematodes	Species	Geographic distribution	Second intermediate hosts; habitats	Population at risk (× 10^6^)
Liver flukes	*Clonorchis sinensis*	China (except for Inner Mongolia, Ningxia, Qinghai, Tibet, Xinjiang), Republic of Korea, Taiwan, Vietnam*	>100 species of freshwater fish; freshwater habitats with stagnant or slow-moving waters (ponds, river, aquaculture, swamps, rice fields)	601.0†
	*Opisthorchis felineus*	Kazakhstan, Russian Federation, Siberia, Ukraine‡	>35 species of freshwater fish; freshwater habitats with stagnant or slow-moving waters (ponds, river, aquaculture, swamps, rice fields)	12.5§
	*Opisthorchis viverrini*	Cambodia, Lao People's Democratic Republic, Thailand, Vietnam‡		67.3¶
	*Fasciola hepatica, Fasciola gigantica*	Altiplano of Bolivia, Cuba, highlands of Ecuador and Peru, Nile delta of Egypt, northern Islamic Republic of Iran, Portugal, Spain‡	Watercress and other water plants (drinking water); irrigation channels, pastures, banks of rivers, ponds, pools	91.1#
Lung flukes	*Paragonimus* spp.	Southwestern Cameroon, China, Ecuador, eastern Nigeria, Peru, the Philippines, Republic of Korea**	>50 species of freshwater crab and crayfish; freshwater habitats with stagnant or slow-moving water (ponds, aquaculture)	292.8††
Intestinal flukes	*Fasciolopsis buski*	Bangladesh, China, India, Indonesia, Malaysia, Taiwan, Thailand‡‡	Water caltrop, water chestnut, water hyacinth, water bamboo, duckweed, water mimosa, water spinach; drainage systems of pig farms, freshwater habitats with stagnant or slow-moving waters	Not known
	*Echinostoma* spp.	China, Indonesia, Malaysia, the Philippines, Republic of Korea, Taiwan, Thailand‡‡	Molluscs, fish, snails and tadpoles; freshwater or brackish habitats with stagnant or slow-moving waters	Not known
	*Heterophyes heterophyes*	China, Egypt (Nile delta), India, Indonesia, Islamic Republic of Iran, Philippines, Sudan, Taiwan, Tunisia, Turkey‡‡	Brackish water fish (mullets, perches, gobies); brackish water habitats	Not known
	*Metagonimus yokogawai*	The Balkans, China, Indonesia, Islamic Republic of Iran, Israel, Japan, Republic of Korea, Spain, Taiwan‡‡	Freshwater (Cyprinid) fish; freshwater habitats	Not known

*References 1 and 18. †Obtained by adding population at risk in China (including Taiwan) (1), Vietnam (10 million; JY Chai, pers. comm.), and Republic of Korea (44% of 2005 population (*17**,**19*). ‡Reference 17. §Obtained by adding 8% of 2005 population in Russian Federation, 1.3% in Kazakhstan, and 2% in Ukraine (*17**,**19*). ¶Obtained by adding 2005 population in the Lao People's Democratic Republic, population at risk in Vietnam (10 million; J.Y. Chai, pers. comm.), and 80% of 2005 population in Thailand (*17**,**19*). #Obtained by adding 23% of 2005 population in Bolivia, 20.6% in Ecuador, 35.3% in Peru, 24.3% in Spain, 44.2% in Portugal, 50.7% in Egypt, 10.8% in the Islamic Republic of Iran, and total 2005 population in Cuba (*17**,**19*). **References 4, 17, and 18. ††Obtained by adding 15.9% of 2005 population in China, 18.9% in Ecuador, 1.4% in Peru, and 14% in Republic of Korea (*17**,**19*). Population at risk in Cameroon estimated at 2.7 million (population of the South and Central province, known foci for paragonimiasis (*20*), estimated at 1.5 million in 1982 [http://www.absoluteastronomy.com/encyclopedia/C/Ce/Centre_Province,_Cameroon.htm]), which we standardized to 2005 (*19*). No estimate was provided for population at risk in eastern Nigeria. ‡‡Reference 4.

Behavioral determinants include unsanitary defecation habits, use of human excreta as fertilizer, and food consumption and cooking habits. In villages near the Nam Pong water resources development project in Thailand, no correlation was found between households with latrines and the extent of opisthorchiasis (*21*). Cooking and food consumption–related determinants are complex and include economic and sociocultural (i.e., beliefs and tradition) factors. Traditional local dishes include raw or partially cooked aquatic products. They are frequently eaten in areas endemic for foodborne trematodiasis and are part of deeply rooted cultures. Examples of typical local fish dishes include raw crab soaked in soy sauce (*ke*-*jang*) in the Republic of Korea, raw drunken crabs and raw grass carp in China, and raw fish (*lab*-*pla* and *plasom*) in Thailand (*1**,**17*). Conversely, in industrialized countries (e.g., Japan), infections are often coupled with foreign travel and eating imported aquatic foods or exotic delicacies (*22*).

Public health interventions, such as chemotherapy, access to improved sanitation, food inspections, and education campaigns to teach proper cooking methods for fish and other potentially contaminated aquatic foods, will affect the pool of parasites. They will also reduce the prevalence of major foodborne trematode infections.

## Geographic Distribution and Population at Risk

Table 1 summarizes the disease-endemic countries and estimated populations at risk for the major foodborne trematodes. For China and Vietnam, we used recent estimates of their at-risk population (*1*) (J.Y. Chai, pers. comm.). For other countries, estimates of at-risk populations have been obtained by multiplying the fraction of a previous estimate of the population at risk provided by an expert committee of the World Health Organization (*17*) by the most recent population figures available (*19*). For example, in 1995, an estimated 19 million people (44%) in the Republic of Korea were at risk for clonorchiasis. Applying the latest United Nations national population statistics (*19*), we estimate that 21 million people are now at risk for infection with *C. sinensis* in the Republic of Korea.

We found that 601 million people are currently at risk for infection with *C. sinensis*, of whom 570 million are in China and Taiwan. *C. sinensis* is also prevalent in Vietnam. *Opisthorchis viverrini* is endemic in Cambodia, the Lao People's Democratic Republic, Thailand, and Vietnam, and *O. felineus* is endemic in the former Soviet Union, Kazkastan, and Ukraine (*17**,**18*). An estimated 67.3 million people are at risk for infection with *O. viverrini* and 12.5 million are at risk for infection with *O. felineus*.

Human fascioliasis is a major public health problem in the Andean countries, western Europe, the Islamic Republic of Iran, Egypt, and Cuba (*16*), with an estimated 91 million people at risk. This figure is half the previous estimate (*17*) because China has not been included in our calculation. Although *F. hepatica* is of considerable veterinary significance in China, human infections are rare (*23*). This finding supports our position not to include this country in the estimate. At least 292.8 million people are at risk for infection with *Paragonimus* spp., with 195 million residing in China. Paragonimiasis also occurs in the Republic of Korea and the Philippines, parts of Africa (eastern Nigeria and southwestern Cameroon), and South America (Ecuador and Peru) (*17**,**18*).

No estimates are currently available regarding populations at risk for intestinal flukes. *F. buski* is common in Bangladesh, China, India, Indonesia, Malaysia, Taiwan, and Thailand (*17**,**18*). Echinostomes have been reported in China, Indonesia, Malaysia, the Philippines, the Republic of Korea, Taiwan, and Thailand (*18*). Among the heterophyids, *Heterophyes heterophyes* and *Metagonimus yokogawai* are the 2 species of greatest medical importance. They are prevalent in the Balkans, China, Egypt, India, Indonesia, the Islamic Republic of Iran, Israel, Japan, the Republic of Korea, the Philippines, Spain, Sudan, Taiwan, Thailand, Tunisia, and Turkey (*4*).

## Aquaculture Development in Trematode-endemic Countries

Aquaculture is the most rapidly growing food sector and global consumption of aquatic products has exceeded that of meat products (*24*). Numerous aquatic products are available at affordable prices to most population segments in the developing world. For ≈1 billion people, these foods provide more than one fourth of their total animal protein supply (*24*). We compiled data on the development of freshwater fish and crustacean production in the past 10–50 years with an emphasis on those countries where *C. sinensis*, *O. felineus*, *O. viverrini*, and *Paragonimus* spp. are endemic. Data were obtained from the Food and Agricultural Organization (http://www.fao.org/fi/default.asp).

**Figure 2:**
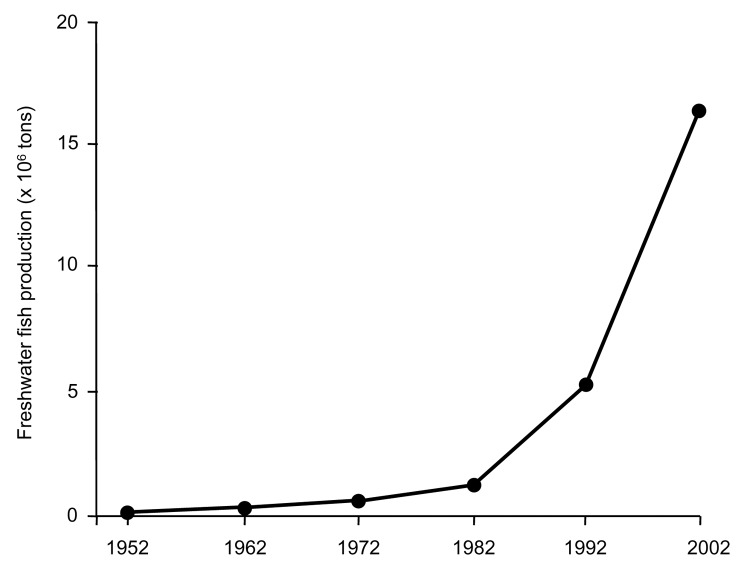
Development of freshwater fish production in China, 1952–2002.

Freshwater fish aquaculture has increased exponentially from an annual production of 136,000 tons in 1952 to 16.6 million tons (Figure 2) 50 years later in China, the Republic of Korea, and Vietnam. Most (97.6%, 16.2 million tons) of this fish is produced in China. In China, the amount of land used for aquaculture has increased by 75% from 2.8 million hectares in 1970 to 4.9 million hectares in 1997 (*7*). Freshwater crustacean production in China has increased 48-fold over the past decade from 9,509 tons in 1992 to 453,696 tons in 2002. These developments are of considerable health concern because fish and crustaceans act as second intermediate hosts of clonorchiasis and paragonimiasis, respectively.

**Figure 3:**
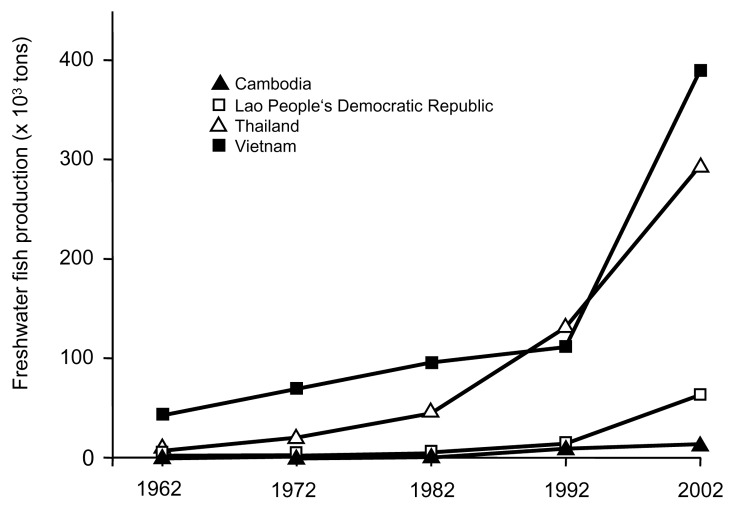
Development of freshwater fish production in Opisthorchis viverrini–endemic countries, 1962–2002.

Figure 3 shows that a large increase in aquaculture has also occurred in the *O. viverrini*–endemic countries of Cambodia, the Lao People's Democratic Republic, Thailand, and Vietnam. In Vietnam, freshwater fish production increased from 41,750 tons in 1962 to 390,000 tons 40 years later (a 9.3-fold increase). Conversely, fish production has decreased by 29.4% in the *O. felineus*–endemic countries of Kazakhstan, Ukraine, and the Russian Federation from 171,542 tons in 1992 to 121,032 tons in 2002.

Available aquaculture statistics are underestimated because small-scale aquaculture or rice field fisheries are not included in officially reported annual production. For example, although the officially reported annual number of inland fish produced in the Lao People's Democratic Republic in 1999 was 25,521 tons, the estimated total figure was 5.9- and 7.8-fold higher (150,000–200,000 tons) (*25*).

Freshwater aquaculture is often largely dispersed, characterized by an informal nature, and usually operated in remote rural areas. Part-time fishing is the rule rather than the exception and, most importantly, the dominant share of the production is eaten within the communities where freshwater fish and other aquatic products are cultivated (*25*).

## Relationship between Foodborne Trematodiasis and Proximity to Freshwater Bodies

We systematically reviewed the literature with an emphasis on proximity of human habitation to any form of freshwater body and its relationship to foodborne trematodiasis. Our search included the National Library of Medicine's Medline, Scielo, Biosis previews, and the Web of Science. We used the following keywords: *Opisthorchis*, *Clonorchis*, *Fasciola*, *Echinostoma*, *Fasciolopsis*, foodborne trematodes, and foodborne trematodiasis in combination with prevalence, water, river, irrigation, dam, aquaculture, pond, and stream. Papers published in English, French, and German were considered. We also included manuscripts in Chinese, Japanese, Korean, and Russian if there was an English abstract of these papers on the aforementioned electronic databases.

Information from 12 studies on the prevalence of foodborne trematode infections in villages located close to freshwater bodies (i.e., rivers, streams, dam reservoirs, and irrigation schemes) and more distant villages is shown in Table 2. Five studies were conducted in the Republic of Korea, 4 in Thailand, 2 in Peru, and 1 in Vietnam. Five studies analyzed *C. sinensis*, 4 analyzed *O. viverrini*, and 2 analyzed *F. hepatica*. *M. yokogawai* was examined in 2 settings.

**Table 2:** Studies comparing the prevalence of foodborne trematode infections in villages close to water bodies with distant villages*

Study site, period (reference)	Population sample	Characteristics of water body	Prevalence	RR (95% CI)
Asillo irrigation area, Peru, 1999 (*26*)	338 school children	500-hectare irrigation area with irrigation canals and drainage channels	*Fasciola hepatica*: 18.8%, 20.3%, 31.3% in 3 schools in irrigation scheme	NA
Kimhae county, Republic of Korea, 1974 (*27*)	1,809	River region	*Clonorchis sinensis*: 72.1% near riverside and 41.3% inland	1.74 (1.57–1.92)
Goyang county, Republic of Korea, 1974 (*27*)	578	River region	*C. sinensis*: 32.7% near riverside and 6.3% inland	5.16 (3.04–8.75)
Hadong Gun, Republic of Korea, 1978 (*28*)	1,163	Rivers and streams	*Metagonimus yokogawai*: 5.4%–90.8% in villages close to river and streams and 4% in village 4 km from river	7.44 (2.83–19.54)
Pohang industrial belt, Republic of Korea, 1989 (*29*)	3,180 employees; 200 for questionnaire analysis	Hyungsai River basin	*C. sinensis*: 52% of infected employees lived near river compared with 27.9% of uninfected employees	1.85 (1.28–2.67)
Okcheon-gun, Republic of Korea, 2000 (*30*)	1,081	Geum-Gang River	*C. sinensis*: 14.2% of inhabitants near river were infected with *C. sinensis* compared with 3.2% of inland residents	4.51 (2.64–7.70)
			*Metagonimus* spp.: 8.4% of inhabitants near river were infected, compared with 1.7% of inland residents	5.01 (2.40–10.46)
Nong Wai irrigation area, Khon Kaen, Thailand, 1974–1975 (*31*)	627 children	Irrigation canal and channels	*Opisthorchia viverrini:* 7.3% in irrigated villages and 3.3% in nonirrigated villages	2.20 (0.87–5.51)
Nam Pong development project, Khon Kaen province, Thailand, 1977–1978 (*21*)	3,183	Reservoir and irrigation scheme	*O. viverrini*: 27.1% in irrigated villages and 17.2% in traditional villages (no irrigation)	1.63 (1.34–2.00)
			*O. viverrini*: 10.8% lakeside and 11.5% in resettlement areas	0.93 (0.66–1.31)
Chonnabot village, Khon Kaen province, Thailand, 1980–1982 (*32*)	4,638; 246 included for incidence calculation	Wide shallow reservoirs that remained dry in 1981–1982	*O. viverrini*: 47% in uninfected individuals becoming positive within 1 year while reservoirs were flooded and 20% during period when reservoirs were dry†	2.17 (1.42–3.29)
18 villages in Nong Khai and Loei provinces, Thailand, 1981–1982 (*33*)	1,259	Khong River and Huang River (flowing water)	*O. viverrini*: 51.7% and 52.6% in villages >5 km from river and 27.9% and 21.7% in villages closer to river	0.47 (0.40–0.56)
12 provinces of Vietnam, 1994–2000 (*34*)	>20,000	Red River delta region	*C. sinensis*: £31% in coastal delta region, 5% in mountainous area, and 16.3% in highlands. *O. viverrini*: highest in urban coastal areas	NA
Mantaro valley, Peru, 2000 (*35*)	206 children	Small streams		Odds ratio 17.22
All studies				2.15 (1.38–3.36)

*RR, relative risk; CI confidence interval; NA, not available. †Incidence values.

**Figure 4:**
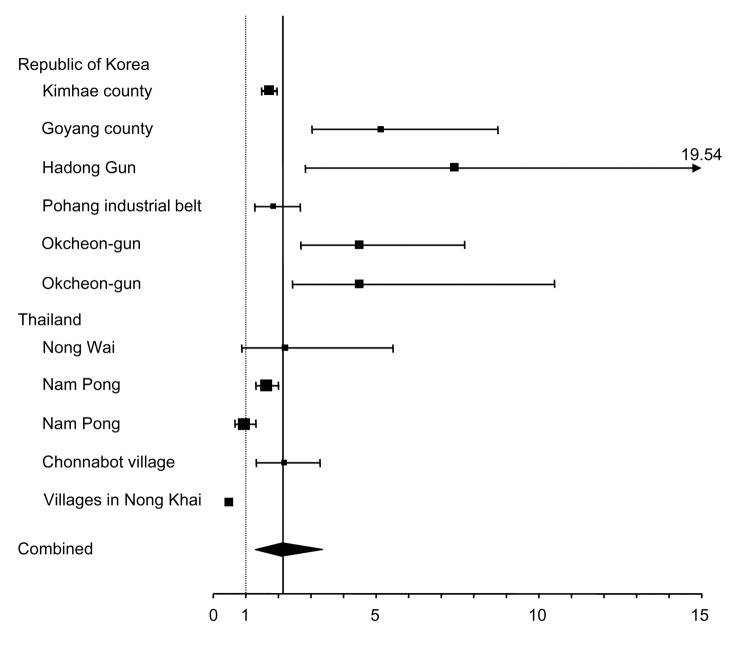
Metaanalysis of studies comparing the prevalence of foodborne trematode infections in villages close to water bodies with distant villages. Values on the x-axis are relative risks. Horizontal bars show 95% confidence intervals. The solid vertical line represents the mean of the combined measure. The diamond represents the combined measure.

Relative risk (RR) calculations were attainable for 10 studies. We calculated RR and 95% confidence intervals (CIs) by using EasyMA software (*36*). A random-effects model was used for calculation of pooled RR because the interventions and conditions in these studies were expected to be heterogeneous (*37*). The results are summarized in Table 2 and shown as Forrest plots in Figure 4. The random summary RR measure was 2.15 (95% CI 1.38–3.36) indicating that risk for infection with foodborne trematodes in villages near freshwater bodies is 2.15-fold higher compared to that farther from the water. In 2 villages on the Khong River and Nam Pong water resources development project in northeastern Thailand, lower prevalences of *O. viverrini* were observed near the river and reservoir compared with villages not using these water sources. These observations can be explained by low snail densities in the dam reservoir (*10*) and the Khong River, the latter because of faster current (*33*).

## Discussion and Conclusions

In an attempt to update the current picture of foodborne trematodiasis, we estimate that 601.0, 293.8, 91.1, and 79.8 million people are at risk for infection with *C. sinensis*, *Paragonimus* spp., *Fasciola* spp., and *Opisthorchis* spp., respectively. In the absence of recent national figures for at-risk populations, number of persons infected, and spatiotemporal distribution of these diseases in most trematodiasis-endemic countries, our estimates should be used judiciously.

Several issues are worth highlighting. First, estimates of persons at risk for major foodborne trematodes are considerably higher than most recent (dating back 10 years) comprehensive estimates. For example, the at-risk population for infection with *C. sinensis* was estimated to be 289 million people in the mid 1990s (*17*), which is less than half of the current estimate. Second, of great concern is the high number (15 million) of *C. sinensis* infections recently reported from China (*1*). Thus, within 10 years the number of *C. sinensis* infections has more than tripled in this country, which warrants in-depth investigations on the underlying causes. Third, it is important to juxtapose these observations with trends observed over the same period, but with an emphasis on soil-transmitted helminthiasis and schistosomiasis. In many parts of Southeast Asia, including China, the number of people infected with *Schistosoma japonicum* and the major soil-transmitted helminths (i.e., *Ascaris lumbricoides*, hookworms, and *Trichuris trichiura*) has decreased (*38**,**39*). These decreases are the result of socioeconomic development and chemotherapy-based illness control programs that largely depend on treatment with praziquantel, albendazole, and mebendazole. The issue of why there was an increase in the number of persons infected with *C. sinensis* when decreases were observed for *S. japonicum* and soil-transmitted helminthes therefore arises. We speculate that aquaculture development is the key risk factor.

Aquaculture is a rapidly growing food sector, mainly in the developing world, and particularly in Asian countries. Development of this sector is of pivotal importance for adequate supplies of food, generation of income, and employment. Different farmed aquatic products are affordable parts of the diet and essential contributors to human health in the developing world (*24*). However, aquaculture development results in ecologic transformations (*40*), and numerous aquatic animal diseases have emerged. Overcrowding and poor environmental conditions have been observed on fish farms, which lead to reduced immunity and higher susceptibility to common diseases (*41*). For example, massive infection with heterophyid metacercariae of aquacultured eels has been documented in Taiwan; dissection showed <3,762 heterophyid metacercariae in a single fish (*42*). In Tasmania, a higher number of trichodinids and cilian protozoan parasites were found in fish raised on farms compared to fish caught in natural bodies of water (*43*).

In reviewing the literature, we found that residents living near bodies of fresh water have, on average, a 2.15-fold higher risk for infections with foodborne trematodes compared to inhabitants of distant villages. Unfortunately, all studies that could be included in our metaanalysis were conducted either in the Republic of Korea or Thailand, and several of these studies date back to the 1970s. Our finding is consistent with previous observations that most of the locally caught aquatic foods are eaten in the communities near freshwater bodies (*17**,**25*). However, with improving transportation and distribution systems, which allow efficient transportation of fish, the amount sold outside the local community is likely to increase considerably. Thus, the spatial distribution of foodborne trematodiasis will change, with an increasing prevalence of these infections in villages where no aquatic products are farmed. We suggest that future studies examine the present spatial distribution of foodborne trematodiasis in Asian countries, compare prevalence of infection in aquaculture workers with other professional groups, and determine the prevalence of parasites in fish raised in aquaculture ponds compared with natural water bodies.

This review emphasizes the important role aquaculture plays in transmitting foodborne trematodiasis. In view of the rapid growth of this food sector, strategies to reduce the current impact of these diseases and to reverse their emergence are mandatory. Safe, efficacious, and inexpensive single-dose oral drugs, such as praziquantel and triclabendazole, are available to treat foodborne trematodiasis and will remain the backbone of control (*5*). To enhance sustainability, chemotherapy should be used with new technologies to ensure food safety, sound health education campaigns for properly cooking aquatic foods, and access to improved sanitation.
